# Effects of Extreme Rainfall on a Dominant Seaweed Are Mitigated by Its Microbiota

**DOI:** 10.1002/ece3.73644

**Published:** 2026-05-14

**Authors:** Alexander H. McGrath, Peter D. Steinberg, Staffan Kjelleberg, Ezequiel M. Marzinelli

**Affiliations:** ^1^ School of Life and Environmental Sciences The University of Sydney Sydney New South Wales Australia; ^2^ Sydney Institute of Marine Science Mosman New South Wales Australia; ^3^ Centre for Marine Science and Innovation, School of Biological, Earth, and Environmental Science University of New South Wales Sydney New South Wales Australia; ^4^ Singapore Centre for Environmental Life Sciences Engineering Nanyang Technological University Singapore Republic of Singapore

**Keywords:** antimicrobial treatments, climate change, extreme weather, holobiont, macroalga, microbiome, reproduction

## Abstract

Extreme weather events are becoming more intense and frequent, driving unprecedented ecological changes globally. The effects of such extreme events can be particularly profound if they affect the performance of habitat‐forming organisms (trees, corals, kelp). Further, the emergence of the “holobiont” concept in biology suggests that these impacts can occur directly on the habitat‐forming “host” and/or via disruption of their associated microbiota. Following a one‐in‐100 year rainfall event along the coast of Sydney, Australia, we examined the effects of rainfall (in the field) and lowered salinity (in the lab) on the performance and reproductive output of a dominant, habitat‐forming intertidal seaweed, *Hormosira banksii*. We then examined the ability of surface‐associated microbes to mitigate host responses to extreme rainfall via microbial manipulations in the field. Extreme rainfall and reduced salinity (< 25 ppt) negatively affected host reproductive output. Manipulative field experiments using a combination of antimicrobial treatments applied once (pulse) or regularly (press) showed that disruption of *Hormosira*'s microbiota after extreme rainfall affected host photosynthesis and, more importantly, inhibited the post‐event recovery of host reproductive output. Press disruption of the host‐microbiota prevented recovery of normal (control) levels of reproductive output and photosynthesis for over 4 months. These experiments demonstrate that host‐associated microbiota can play a significant role in mediating responses of habitat‐forming seaweeds to extreme weather events, with consequences for key components of fitness. Given the increased frequency of flooding and storm events experienced by many systems, the microbiome may provide a key role in influencing habitat resilience to stress.

## Graphical abstract

Extreme weather events, such as intense rainfall, can severely reduce the reproductive output and photosynthetic performance of habitat‐forming seaweeds like Hormosira banksii. This study shows that disrupting the seaweed's microbiota after such events further hinders recovery, highlighting the crucial role of host‐associated microbes in maintaining ecosystem resilience under increasing climate stress.

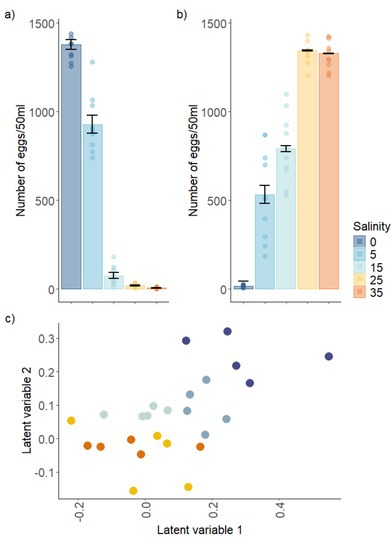

## 1 Introduction

Climate change is driving an increase in the frequency and severity of extreme weather events such as heatwaves, droughts and rainfall (Hoegh‐Guldberg and Bruno 2010; Masson‐Delmotte et al. 2022; Solomon et al. 2007). The ecological consequences of such extreme events can be severe, particularly where habitat‐forming species are affected, because this can lead to cascading effects on the ecosystems they underpin (Goulding et al. 2016; Poloczanska et al. 2016; Sarà et al. 2021). Heatwaves are among the most studied extreme events, primarily because of the ease of measurement and the recent occurrence of several heatwaves that have strongly impacted the ecological structure, functioning and resilience of habitats across realms, such as coral reefs (Vompe et al. 2024), macroalgal forests (Smale et al. 2019; Straub et al. 2022), estuaries (Tassone et al. 2022), forests (Hoover et al. 2014) and grasslands (Teuling et al. 2010). However, other extreme events which are influenced by a changing climate, such as more severe storms and associated intense rainfall, are also becoming prominent (Fong et al. 2020; Sabater et al. 2023). For example, intense rainfall events can have a substantial effect on the structure (O'Gorman et al. 2012) and functioning of ecosystems ranging from rivers (Espinoza et al. 2022; Sabater et al. 2023) and grasslands (Fay et al. 2008; Grant et al. 2014) to coastal habitats (Malan et al. 2024). The impacts of extreme rainfall can be due to changes in rainfall per se (i.e., increased water flow or freshwater input), but also due to synergistic effects which are characteristic of extreme rainfall events, such as nutrient enrichment and chemical pollution (Kuntz et al. 2005; O'Connor et al. 2015; White et al. 2018). Extreme storms and associated rainfall events can affect the physiology and ecology of habitat‐forming species, negatively affecting their performance and survival (Fay et al. 2008; Fong et al. 2020; Grant et al. 2014). Effects such as cellular damage (Davison and Pearson 1996), reduced growth rates (Kroeker et al. 2010), reduced recruitment (Deysher and Dean 1986), changes in ecological interactions (Grant et al. 2014) and survival have commonly been reported for habitat‐forming organisms ranging from plants (both terrestrial and aquatic) (Death et al. 2015; Fay et al. 2008; Grant et al. 2014), to sponges (Pita et al. 2018; Ribes et al. 2016), corals (Fong et al. 2020) and macroalgae (Straub et al. 2022; Veenhof et al. 2022; Wernberg et al. 2013).

One largely unexplored aspect of how habitat‐formers respond to, and recover from extreme events is the role of their associated microbiota (but see Augé 2001, Fong et al. 2020, Ali et al. 2022 for exceptions). Plants, seaweeds, corals and other habitat‐formers exist in nature as holobionts, that is, the eukaryotic host and its associated microbial community, or microbiota (Laforest‐Lapointe et al. 2017; McFall‐Ngai et al. 2013; Rosenberg and Zilber‐Rosenberg 2018). Stressors such as rainfall may act directly on host physiology, but for holobionts, they may also act via disruption of the host's microbiota. There is strong evidence that microbiota associated with eukaryotic hosts are important for their normal development and function (Laforest‐Lapointe et al. 2017; Li et al. 2022; Marzinelli et al. 2024; McGrath et al. 2024), influencing host properties or processes such as morphogenesis (Matsuo et al. 2005; Ortíz‐Castro et al. 2009), photosynthesis (Qiu et al. 2019), nutrient cycling and provision (Minich et al. 2018; Pfister et al. 2019) and defence against pathogens (Li et al. 2022; Longford et al. 2019; Sarma et al. 2015). Thus, if the increase in frequency and intensity of extreme events leads to significant disruptions of microbiota, this in turn may affect the host—an indirect effect rarely explored.

Beyond these established functional roles, host‐associated microbiota may also influence life historical or demographic processes—that is, components of fitness—that are fundamental for the establishment or persistence of habitat‐forming organisms. However, we still know little about how microbiota affect the core components of holobiont fitness—survivorship and reproduction—particularly for habitat‐formers and in the field (Garbary and London 1995). Although understanding the effects of microbiota on survivorship on long‐lived organisms can be challenging (except for acute events), reproductive output, that is, the number of viable offspring produced by an individual, is often a useful proxy of fitness (Alif et al. 2022; Hamel et al. 2010; McCoy et al. 2020) and can provide insights on the persistence and re‐establishment of populations after a severe disturbance. We used reproductive output, as well as a physiological measure of photosynthesis, in this study as a metric to quantify responses of a habitat‐forming holobiont to an extreme rainfall event.

Within the marine environment, macroalgae are often more susceptible to extreme rainfall than other habitat‐forming species because of their sensitivity to nutrient additions from terrestrial run‐off (Bellgrove et al. 2010, 2017; Fong et al. 2020; Worm et al. 1999), relative intolerance to rapid salinity changes (Fong et al. 1996; Takolander et al. 2017) and inability to move to more favourable conditions. However, fully understanding the effects of extreme weather events on macroalgae is challenging because of the numerous life history stages and transitions upon which environmental stressors can act (Schiel and Foster 2006). Stress tolerance, and thus effects of stressors, may vary across different life history stages within a species (Fain and Murray 1982; Ladah and Zertuche‐González 2007). *Macrocystis pyrifera* provides a clear example of this variability across ontogeny, with temperature stress negatively affecting spore production (Buschmann et al. 2004), germination (Buschmann et al. 2004), recruitment (Deysher and Dean 1986) and sporophyte growth (Rothäusler et al. 2009, 2011) in some, but not all, places within the distribution of the species (Deysher and Dean 1986; Muñoz et al. 2004). Thus, incorporating multiple life history stages into studies of the impact of extreme events is critical to better understand how individuals or populations of habitat‐forming organisms will respond.

To test whether a holobiont's microbiome could mitigate response to extreme weather events, we investigated the response of *Hormosira banksii*, a dominant seaweed on Australian intertidal rocky shores, to extreme rainfall events and experimentally tested whether those responses were mediated by its surface‐associated microbiota. Specifically, we hypothesised that extreme rainfall would have significant effects on *Hormosira*'s reproductive output. If effects of rainfall on the host were primarily due to freshwater (as opposed to other rainfall‐associated stressors such as nutrients or toxicants), we predicted that exposure of hosts to freshwater levels in the laboratory, similar to those observed in rainfall events in the field, would lead to lowered reproductive output, comparable to that observed during rainfall events in the field. We further examined whether the host‐associated microbiota mediated host responses to, and recovery from, extreme rainfall. We hypothesised that (i) disruption of the host's microbiota would reduce the ability of the host to recover its reproductive output and photosynthesis after extreme rainfall events; and (ii) such responses would be strongest where the host‐microbiota was subjected to chronic, press disruption, as opposed to one‐off (pulse) disruption. This approach focuses on identifying functional links between microbiota and host performance, rather than resolving the specific mechanisms involved.

## 2 Methods

### 2.1 Extreme Rainfall Events

Rainfall data were collected from the Bureau of Meteorology online data portal (https://www.bom.gov.au/climate/data/) using the Sydney Airport weather station (Sydney, NSW, Australia; 151.17 °E, 33.95 ^o^S). Alongside this remotely sensed data, during each monthly sampling (described below), salinity was measured in the rock pools at Cape Banks Aquatic Reserve during low tide using a refractometer (Extech, *n* = 5).

### 2.2 Effect of Extreme Rainfall on *Hormosira*'s Reproductive Output

To determine the effect of extreme rainfall on reproductive output of *Hormosira banksii* (*Hormosira* hereafter), 20 female *Hormosira* of approximately 1.3 ± 0.5 cm in diameter and 12.5 ± SE 0.4 cm in length, ~1 m apart were collected from the rocky platform at Cape Banks Aquatic Reserve, Sydney (33°59′55.3″S 151°14′53.6″ E) during low tide each month from October 2021 through to March 2023 (*N* = 340; Specific dates within Table S2). Female individuals can be easily identified from males in the field by examining the reproductive structures. Males of this species have orange conceptacles (due to the colouration of the antherozoids), whereas female conceptacles are olive in colour. Spawning was induced by placing algae into a refrigerator at 4°C for 12 h (as per Dimartino et al. 2016; Forbes and Hallam 1978; Figure S1) and then into individual containers with no water and under a high LUX (~500 Lux) lamp for 2 h at room temperature. This laboratory technique does not directly mimic natural spawning but shocks the algal individual into gamete release (Dimartino et al. 2016). Gametes were then washed from the individuals with 50 mL of artificial filtered seawater (AFSW), and 5 subsamples of 5 mL were used to count the number of eggs released by each individual female (*n* = 20 per month).

### 2.3 Effect of Salinity on *Hormosira*'s Reproductive Output

**FIGURE 1:**
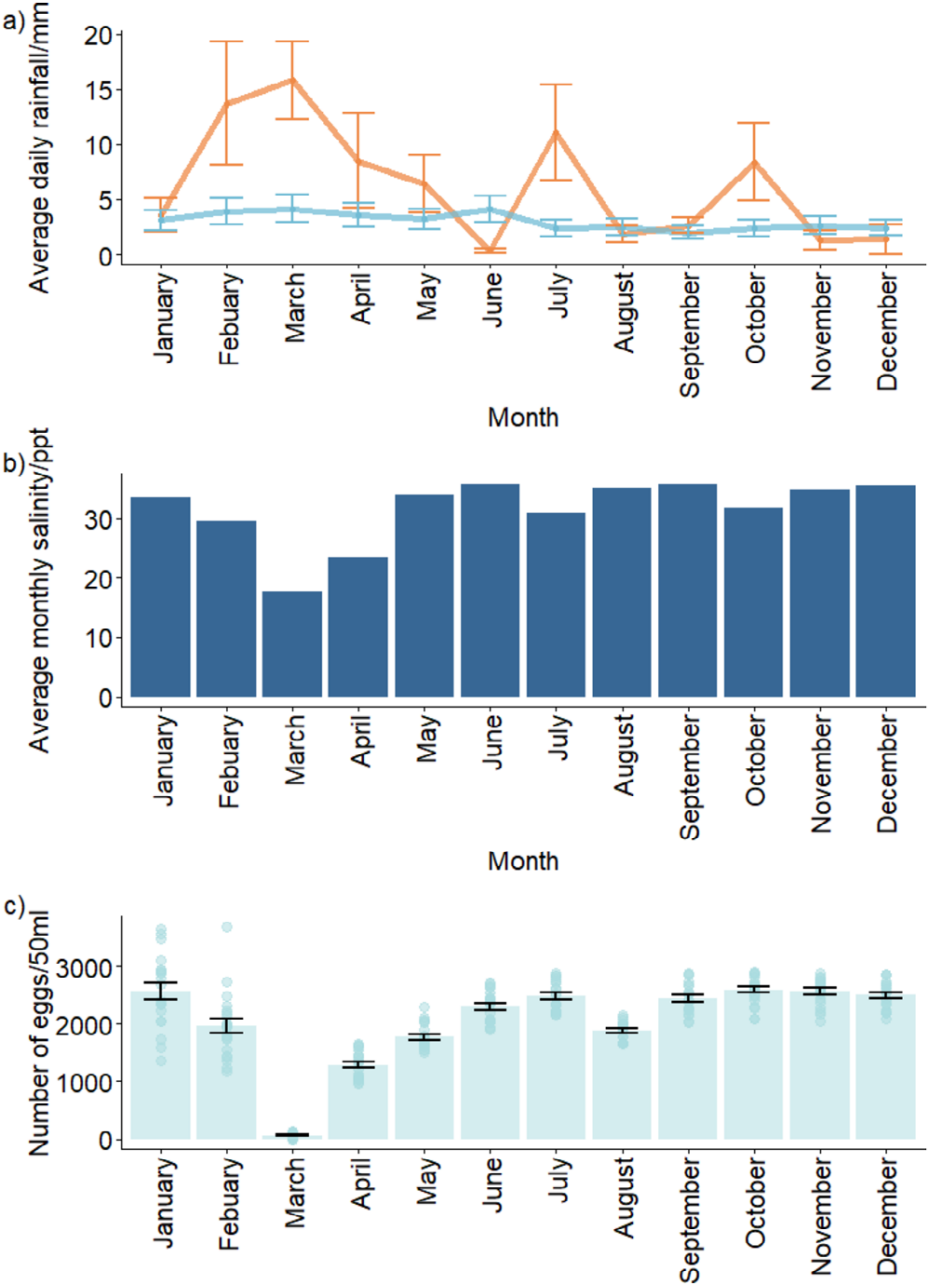
Rainfall, salinity and *Hormosira*'s reproductive output in the field. (a) Average daily rainfall/mm. Orange line is 2022, blue line is average from 1972 to 2022; data are mean (±SE). (b) Average monthly salinity over 2022. (c) Number of eggs released per individual at each monthly sampling time, data are means ± SE (*n* = 20).

To directly test the effect of lowered salinity on reproduction in *Hormosira*, 80 female individuals (approximately 1.3 ± 0.5 in diameter; 12.3 ± SE 0.6 in length) separated by ~1 m from each other were collected at Cape Banks Aquatic Reserve during low tide in March 2023, ~1 year after the most extreme rainfall event during our study, outside of the heavy rainfall periods of 2022. Individuals were transported in seawater within 30 min to The University of Sydney where they were placed into a recirculating aquarium for 24 h to acclimate. 15 individuals were then placed into separate 0.5 L tanks and randomly assigned to each of five salinity treatments to represent the range of salinity experienced in the field (see above and Figure 1A): Freshwater (0 ppt), 5 ppt, 15 ppt, 25 ppt and 35 ppt. To avoid rapid osmotic stress to the individuals, salinity levels were decreased to the target level over the course of 1 h (as per Davis et al. 2022). Individuals were left in each of these treatments for 24 h. To see whether any eggs were released as a stress response to the salinity treatments, whereas in the tanks, the number of eggs released into each tank was counted by taking 5, 5 mL subsamples of the tank water. Spawning was then induced (via placing algae in the fridge 4°C, as above) in 10 individuals from each treatment to give the number of eggs released per 50 mL of AFSW. To ensure that the individuals were fecund (i.e., they had not spawned prior to being placed in tanks), an additional 20 individuals were also collected from the field site as part of the monthly spawning (as described above; ‘Effect of extreme rainfall on *Hormosira*'s reproductive output’) and spawned as described above. The remaining 5 individuals from each treatment were then sampled to characterise their surface‐associated bacterial communities (described below).

### 2.4 Effect of Disruption of Surface Microbiota on Recovery of Host Function

210 female *Hormosira* individuals of similar size (approximately 1.2 ± 0.6 in individual thallus diameter and 11.9 ± SE 1.1 in length) separated by ~1 m were tagged at Cape Banks Aquatic Reserve during low tide on the 24th of March 2022 during the extreme rainfall event, but on a day without heavy rain. Algae were rinsed with AFSW for 30 s to remove loosely attached epiphytes and then subjected to one of 6 treatments, on the basis of the methods of McGrath et al. (2024): (1) pulse microbial disruption via exposure to an antibiotic combination containing Penicillin (100 mg/mL), Neomycin (100 mg/mL) and Rifampicin (50 mg/mL) applied once for 4 h; (2) pulse microbial disruption via exposure to iodine (povidone‐iodine Betadine) applied once for 4 h; (3) a pulse procedural control (AFSW applied for 4 h); (4) undisturbed control; (5) press microbial disruption via exposure to iodine applied for 4 h every month; (6) press procedural control (AFSW applied for 4 h every month). To ensure the treatments did not spill over and affect the surrounding algal individuals, a ring of sponges was placed around each individual while the treatment was applied. The ring was then removed after 3 h as the tide encroached, and the treatment was rinsed off the algae by the tide. Samples (*n* = 5 per treatment) were collected at day 0 (before treatment application), 7, 21, 35, 60, 90, and 120.

Host performance following disruption of surface microbiota was quantified in several ways. Maximum photosynthetic quantum yield (after dark‐adapting individuals for 15 min) was measured using a pulse amplitude modulated (PAM) fluorometer (Walz, Germany). PAM was used because previous work has shown it to be a metric that generally reflects the condition of photosynthetic hosts under stress (Qiu et al. 2019; Straub et al. 2019). Egg development was estimated by taking thin sections of the third vesicle from the apical end of the longest frond of each individual *Hormosira*. To do this, fronds were placed in 80% ethanol for 24 h before embedding in paraffin. Sections were then taken on a microtome set to 7um, stained with Tylenol blue (0.4%) and the number of developing eggs counted in the sexual conceptacles under a light microscope. Reproductive output was quantified for each individual as the number of eggs released per ml of AFSW (as detailed above).

To characterise the community structure of the host‐associated microbiota, the surface of each alga was swabbed for 30 s with a sterile cotton swab to collect surface‐associated microbiota at each of the seven time‐points described above, following established methods (Marzinelli et al. 2015; McGrath et al. 2024; Qiu et al. 2019). Swab controls (i.e., treated similarly to those for swabbing algae except no algae were swabbed) were used to test for contamination during processing. Swabs were immediately placed into sterile cryogenic tubes and placed in liquid nitrogen and then stored at −80°C until DNA extraction.

### 2.5 DNA Extraction and Sequencing

Microbial DNA was extracted from each swab sample in a randomised order to avoid introducing any bias due to order and time of processing, using a Powersoil DNA Isolation kit (Qiagen) following the manufacturers' protocol. DNA extracts were quantified using spectrophotometry (Nanodrop 1000) and stored at −20°C until sequencing.

The extracted DNA samples were amplified with polymerase chain reaction (PCR) using the 16S primers 341 (F) (5′‐CCTACGGGNGGCWGCAG‐3′) and 805 (R) (5′‐GACTACHVGGGTATCTAATCC‐3′), containing the V3‐V4 regions of the bacterial and archaeal 16S rRNA gene (Klindworth et al. 2013). The PCR conditions involved a pre‐heating step to 95°C for 3 min followed by 35 cycles of 95°C for 15 s, 55°C for 1 min and 73°C for 30 s. Both positive (with known DNA sequence) and negative controls (nuclease‐free water, control swabs) were used. The negative controls did not amplify DNA, suggesting no contamination on swabs or during extraction and amplification. Agarose gel electrophoresis and Nanodrop 1000 were used to ensure the quantity and quality of the amplicons before they were sent for sequencing via the Illumina MiSeq i100 platform at the Ramaciotti Centre for Genomics (UNSW, Sydney).

### 2.6 Bioinformatics

Raw sequences (10,418,632) were received from the sequencing centre as demultiplexed pair‐ended sequences per sample. UNOISE was then used to remove chimeras and produce amplicon sequence variants (ASVs), that is, ASVs at a unique sequence level (0% distance) (Edgar 2016). DADA2 was used to map the original reads back to ASVs, generating a table of 17,387 ASVs. ASV sequences were searched with BlastN against the SILVA SSU Ref NR99 database for taxonomic classification to classify and remove chloroplasts; the Global Taxonomy Database (GTDB) was then used for taxonomic assignment. Singletons and low‐abundance taxa (< 0.01% of reads) were removed from the dataset for statistical analyses, resulting in 13,210 microbial taxa in 86% retained reads.

### 2.7 Estimation of Absolute Bacterial Abundance

Total abundance of the 16S rRNA gene was quantified for each sample by qPCR using the primers 341F/805R developed by Thijs et al. (2017). Gene amplification and analysis were performed using the QuantStudio 3 thermocycler (Thermo Fisher with PrimeTime Gene Expression Master Mix, Integrated DNA Technologies) and associated software. The reaction conditions for amplification of DNA were 50°C for 2 min, 95°C for 10 min and 40 cycles of 95°C for 15 s and 60°C for 1 min. The final gene copy number per sample was corrected for the total extraction volume, the surface area and the dilution factor and DNA yield per sample (see Nappi et al. 2022 for further details) and was used to estimate absolute abundances of ASVs and inoculants.

### 2.8 Statistical Analyses

To test for extreme events, defined as rare events (> 95th probability percentile) for the area and time of year (IPCC 2023), each month in 2022 was ranked against the previous 50 years of rainfall data on a month‐by‐month basis. To determine which months (within 2022) had the greatest amount of daily rainfall, we used a one‐factor analysis of variance (ANOVA) with the fixed factor of month (12 levels). Furthermore, to determine how salinity changed during extreme rainfall events, salinity was taken from the Bio Oracle V3 database and ranked against previous years (Assis et al. 2024). We characterised the rainfall in March 2022 as an extreme event as the rainfall was greater than the 99th percentile of monthly rainfall on record (Malan et al. 2024), with June and October both being present within the 90th percentile.

To determine whether rainfall, salinity and *Hormosira* reproductive output in the field differed among months in 2022, an ANOVA was run with the factor month for each of these response variables in R (v4.0.3). A Spearman's correlation was also conducted to determine whether there was a monotonic relationship between rainfall and reproductive output, and linear and robust linear regression (MASS::rlm) were used to evaluate the influence of extreme observations and assess the sensitivity of results to outliers (Figure S3).

To test for effects of microbial disruption on host performance and reproduction in the field experiment (PAM, number of developing eggs in *Hormosira* fronds, number of eggs released), we used a two‐factor ANOVA with the orthogonal factors treatment (fixed, 6 levels) and time (fixed, 7 levels) using the R GAD package. To meet the model's assumption of homogeneity of variance, the response variables (a) number of developing eggs and (b) eggs released were square‐root transformed. Post hoc contrasts were run on significant interaction terms, or significant main effects (when no interactions were present) with more than two levels, using emmeans in R, using a Tukey HSD to correct for multiple tests. (Lenth et al. 2019).

For bacterial data, to account for uneven sequencing depth among samples, data were normalised using total 16S rRNA reads per sample of the v3‐v4 region, which was calculated using qPCR (Nappi et al. 2022). Alpha diversity measures of richness (i.e., number of unique sequences) and Simpson's diversity index were calculated using the ‘vegan’ R package (Oksanen et al. 2013), and differences between treatments (fixed, 6 levels), time (fixed, crossed, 7 levels) and their interaction were examined using an ANOVA in the R GAD package (Sandrini‐Neto et al. 2010), as described above. For the salinity lab experiment, bacterial data were processed and analysed in the same way, but with the single factor salinity (fixed, 5 levels).

To determine differences in the structure of the host‐associated bacterial assemblages, the normalised ASV data were analysed using permutational multivariate analysis of variance (Anderson and Walsh 2013) in the R vegan package (Oksanen et al. 2013) with the fixed factors treatment, time and their interaction (for the field experiment), or with the fixed factor salinity (for the lab experiment), as above. These multivariate analyses were based on Bray–Curtis dissimilarities between sample pairs calculated on square‐root transformed absolute (qPCR normalised) abundances of ASVs. For significant main effects or significant interactions, pairwise comparisons were performed using the pairwise.adonis.2 function in the pairwise.adonis package (Martinez Arbizu 2020).

To determine which bacterial taxa differed the most among treatments and times and their interaction (for the field experiment), or among salinity levels (for the lab experiment), we used multivariate generalised linear models (GLMs) using the R package ‘mvabund’ (Wang et al. 2012) assuming a negative binomial distribution to account for over‐dispersion of sequence counts. Generalised linear latent variable models (GLLVMs) were used to visualise the bacterial community structure (Niku et al. 2019) with model fit being assessed by log‐likelihood ratios and checking residuals.

## 3 Results

### 3.1 Effect of Extreme Rainfall on Reproductive Output

Rainfall in Sydney during 2022 was 209% greater than the average of all years since data has been collected (164 years). Rainfall significantly varied over the year, with March 2022 recording the highest monthly rainfall (> 15 mm/day; daily max. 156 mm; 99th percentile in data of previous 50 years; ANOVA, F_11,288_ = 431.8, *p* < 0.001; Figure 1a; Table S1). Rainfall was negatively associated with *Hormosira*'s reproductive output, with thalli in March 2022 having a significantly lower reproductive output than all other months (ANOVA, F_11,228_ = 367.5, *p* < 0.001; Figure 1a–c). April had significantly lower reproductive output than all other months except March (Figure 1c). February, May, and August had significantly lower reproductive output than the remaining months, other than March and April (Figure 1c; Table S2). Rainfall (both daily mean and monthly sum) and reproductive output were negatively correlated (Spearman's rho = −0.73 and −0.51 for mean and sum, respectively, *p* < 0.001). These relationships was mainly driven by rainfall in March (> 15 mm/day); removing data from March led to a non‐significant correlation, suggesting a threshold effect (rho = −0.044, *p* = 0.52 and rho = −0.031, *p* = 0.68, for mean and sum, respectively), whereas robust regression confirmed a strong negative effect of rainfall (*β* = −1224.0 ± 103.9 SE, *p* < 0.001, 95% CI [−1428.7, −1019.3], Figure S3) Salinity significantly varied over the year, with March 2022 recording the lowest average salinity (~18 ppt; 95th percentile in data of previous 10 years; ANOVA, F_11,48_ = 682.19, *p* < 0.001 for in situ data).

### 3.2 Effect of Salinity on Reproductive Output and Host‐Associated Microbiota

**FIGURE 2:**
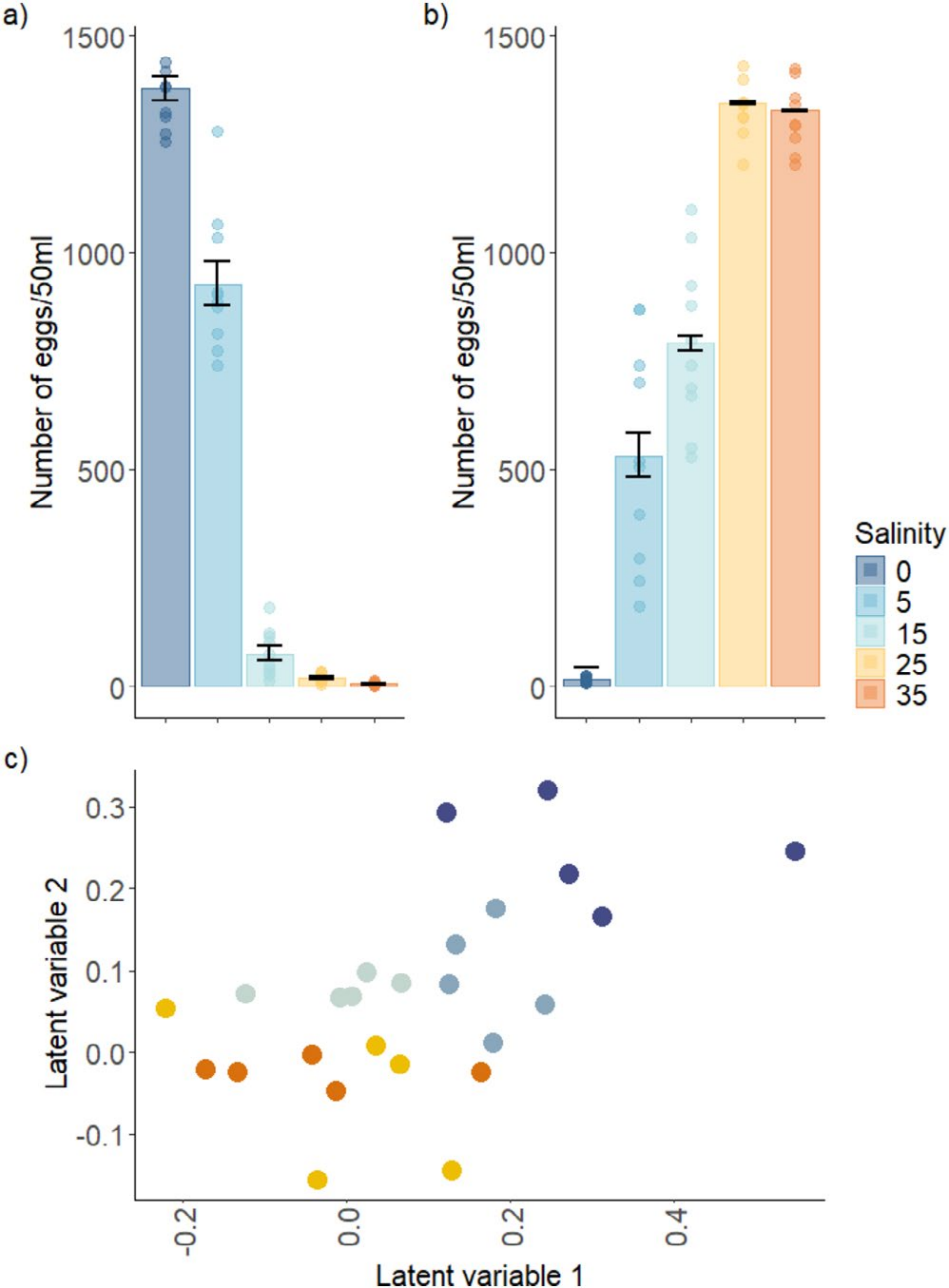
Effect of lowered salinity on *Hormosira*'s reproductive output and microbiota. (a) Number of eggs released in tanks during exposure to salinity stress. Data are means ± SE (*n* = 10). (b) Number of eggs released during subsequent induced spawning at the end of the exposure to the salinity treatments. Data are means ± SE (*n* = 10). (c) Ordination on the basis of a GLLVM model on bacterial community structure associated with *n* = 5 independent replicates. Treatments were Freshwater (0 ppt, Navy Blue), 5 ppt (Blue), 15 ppt (Light Blue), 25 ppt (Yellow), 35 ppt (Orange).

Exposure to lower salinity led to higher numbers of eggs spawned by *Hormosira* in the laboratory into the treatment tanks (ANOVA, F_4,45_ = 376.8, *p* < 0.001, Figure 2a; Table S3). Freshwater (0 ppt) caused the highest level of stress spawning (98% of eggs present per conceptacle), that is, spawning induced in response to induced salinity stress rather than natural spawning typical to the life history of the species (in response to light and temperature), which was significantly different from spawning in all other salinity levels (Figure 2, Table S3). 15 ppt had significantly higher levels of stress spawning than 25 and 35 ppt (which did not differ from each other), but lower than freshwater and 5 ppt (Table S3). The inverse relationship was observed when considering the eggs released once the individuals were spawned using the same methods as in the field experiment. That is, high salinities (25 and 35 ppt) had significantly greater numbers of eggs spawned than lower salinities (ANOVA, F_4,45_ = 136.6, *p* < 0.001, Figure 2b; Table S4).

Salinity had a significant effect on the community structure of *Hormosira*'s microbiota (pseudo‐F_4,45_ = 3.654, *p* < 0.001, Figure 2c; Table S4). Microbiota on algae in the freshwater (0 ppt), 5 ppt and 15 ppt were significantly different from each other and from 25 ppt and 35 ppt, which did not differ from each other (Figure 2c).

GLM analyses identified 127 ASVs associated with *Hormosira* (approx. 1.5% of a total of 8256 ASVs) whose abundances significantly change within the different salinities. Of those, we detected a significant negative effect of salinity on the abundance of 82 ASVs in the families *Cyanobacteriales, Microtrichales*, *Vibrio, Verrucomicrobiales, Rhodobacteriales* and *Rhizobales*, with decreases in abundance ranging from 13% to 34% in 15 ppt and 5 ppt and the greatest decreases (~84%) in 0 ppt.

### 3.3 Effect of Microbial Disruption on Host Reproductive Output and Photosynthesis

**FIGURE 3:**
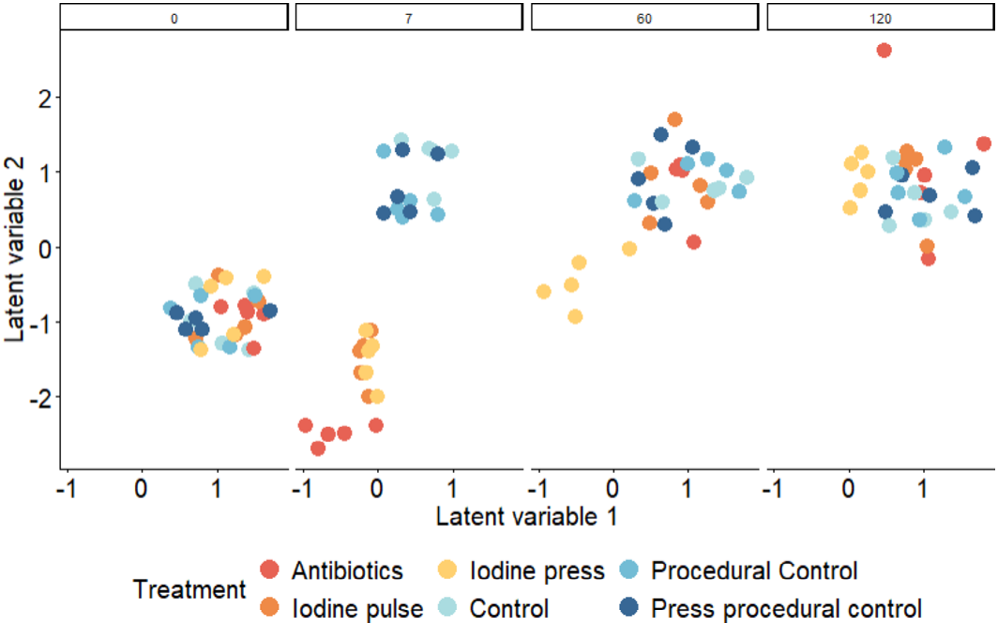
The structure of the bacterial community associated with *Hormosira* following microbial disruption in the field. Ordination on the basis of a GLLVM model with each panel indicating a different sampling day throughout the experiment, with *n* = 5 independent replicates of each of the 6 host treatments: Antibiotics (Red), Iodine pulse (Orange), Iodine press (Yellow), Control (Light blue), Press procedural control (Blue), Pulse procedural control (Navy blue). The full time series plot is found within Figure S2.

Antibacterial treatments used to disrupt the surface‐associated microbiota of *Hormosira* resulted in significant differences in the host's surface microbiota across treatments and times (pseudo‐F_30,168_ = 6.903, *p* < 0.001, Figure 3, Table S5). Antibiotics (AB2) and iodine caused significant changes in the bacterial community structure compared to controls from day 7, a change that lasted until day 60 (Figure 2; Table S6). Antibiotics (AB2) had a different effect on the microbial community structure than iodine, for both pulse and press application, from days 7 to 60 (Table S4), when compared to controls. Press application of the iodine treatment caused significant and lasting effects compared with controls for the entire experimental period of 120 days, and was significantly different from all treatments from day 60 (Figure 3).

The disruption of the surface microbiota with antibiotics (AB2) and iodine caused significant decreases in ASV richness and Simpson's diversity, which recovered to control levels over time (Table S6). Antibiotics caused a greater decrease in ASV richness than iodine, although this decrease recovered to control levels by day 60 (Table S6). The only treatment which remained significantly different from all others at day 120 was that using repeated application of iodine (press; Table S6).

GLM analyses identified 348 ASVs associated with the host (approximately 2.6% of the total 13,210 ASVs) whose abundances differed significantly between treatments. Of these ASVs, we found a significant negative effect of treatment on abundances of 32 ASVs in the families *Pleurocaspa, Marinomonas, Geminocystis, Vibrio*, and *Flavobacteriales*, with decreases ranging from 18% to 73% in the disruption treatments (AB2, Betadine pulse and Betadine press).

**FIGURE 4:**
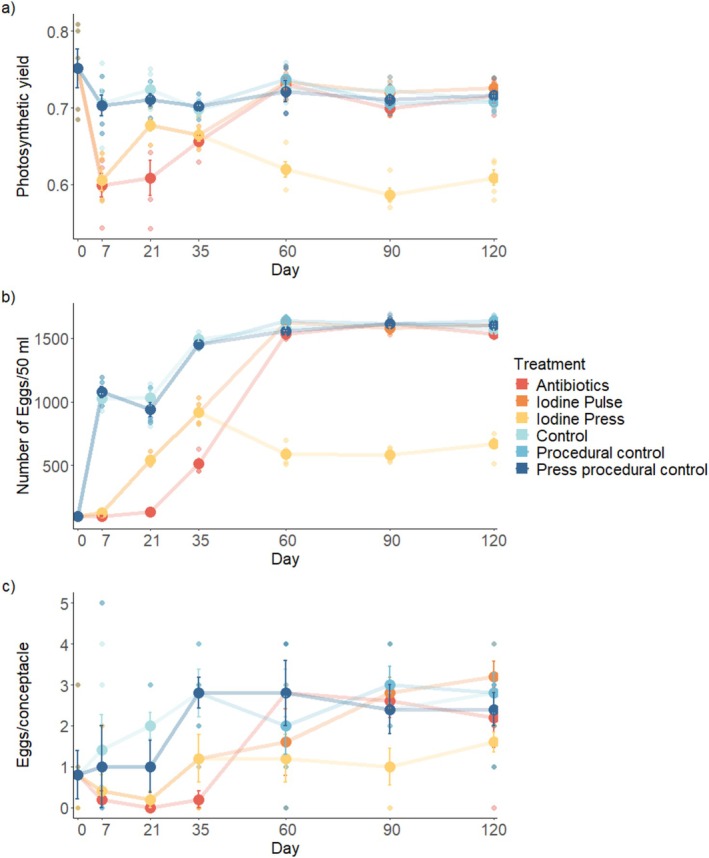
Host response variables over time during the field experiment. (a) Host photosynthetic yield. (b) Reproductive output. (c) Number of developing eggs in each vesicle. Host treatments were: Antibiotics (Navy Blue), Control (Blue), Iodine pulse (Light Blue), Iodine press (Yellow), Press procedural control (Orange), Pulse procedural control (Red). Data are means ± standard error; *n* = 5.

Host reproductive output in the field was significantly affected by these manipulations to the microbiota, with the effect varying over time (ANOVA, F_30,164_ = 105.71, *p* < 0.001; Figure 4b; Table S7). Reproductive output in thalli subjected to pulse treatments recovered to pre‐freshwater stress levels by day 60 (Figure 4b; Table S7). However, press disruption when applied to thalli caused significantly lower reproductive output relative to controls from day one until day 120 and relative to other disruption treatments from day 60 to day 120 (Figure 4b; Table S7). Overall, across all treatments, host reproductive output also increased over time after the initial freshwater stress at the start of the experiment (Figure 4b; Table S7).

The number of developing eggs generally increased over the course of the experiment following the peak in rainfall in March 2022 (Figure 4c, Table S8). Microbial disruption with AB2 and iodine caused a significant lag in the recovery of *Hormosira*'s egg development (Figure 4c). Individuals treated with antibiotics and iodine had significantly lower numbers of developing eggs than controls until day 60, where there was no significant difference (Table S8).

Disruption to the host microbiota caused significant negative effects on host photosynthetic efficiency (ANOVA, F_5,168_ = 54.3, *p* < 0.001; Figure 4A; Table S9). Hosts whose microbiota were treated with pulse disruptions recovered over time, and by day 60, there was no significant effect of these treatments (Table S9). Press treatments, however, caused significant effects on host photosynthetic efficiency, which lasted the entire experimental period (120 days; Figure 4a). Antibiotics had a greater negative effect on host photosynthetic efficiency than iodine when applied in a pulse treatment, which lasted until day 60, after which individuals from antibiotics and iodine treatments returned to control levels (Figure 4a; Table S9).

## 4 Discussion

Extreme events can cause significant, negative impacts on host function and physiology with lasting effects on host persistence and survival (Garrabou et al. 2022; Kuntz et al. 2005). In this study, a one‐in‐100‐year rainfall event resulted in significant impacts on the reproductive capacity of a dominant habitat‐forming seaweed and the extent of the impact was in turn significantly affected by bacteria associated with the seaweed host, influencing host recovery following extreme rainfall events. Disruption of *Hormosira*'s microbiota with antibiotics and antiseptics (iodine) in single application “pulse” treatments took 2 months longer to recover photosynthetic performance and, importantly, reproductive capacity following the extreme rainfall event than controls. Once the microbiota recovered, host photosynthesis and reproductive output also recovered. Conversely, for treatments with repeated microbiota disruption, or “press” treatment, the surface microbiota did not recover, and there was also no recovery of host photosynthesis or reproductive output. This experimental work thus provides insights into the importance of the host‐associated microbiota in mitigating the effect of extreme events on host performance and reproductive output, an important component of the host's fitness.

### 4.1 Extreme Events Within Chronically Stressed Environments

Global environments are experiencing an increase in the frequency and severity of extreme weather events such as heatwaves, droughts, fires and heavy rainfall (Hoegh‐Guldberg and Bruno 2010; Masson‐Delmotte et al. 2022; Solomon et al. 2007). Although heatwaves, droughts and fires are well‐documented stressors, extreme freshwater flooding is emerging as a critical yet underappreciated driver of ecological change. Flood events can drastically alter hydrological regimes and introduce acute physical and chemical disturbances, with cascading impacts on ecosystem structure and function (Balser et al. 2002; Grant et al. 2014; Talbot et al. 2018; Tassone et al. 2022).

As the frequency and intensity of extreme events increase globally, organisms are often experiencing multiple stressors that can create pulse (e.g., acute, extreme events) and/or press disturbances (prolonged, chronic stress) (Glasby and Underwood 1996; Nielsen et al. 2012). Experiments that integrate press disturbance with periodic extreme pulse disturbances, or experiments that combine asynchronous pulse disturbances from different stressors, may best reflect emerging climate realities, particularly in systems where background stress is becoming the norm and extreme events are increasingly layered upon it. For instance, in grassland ecosystems, press drought disturbances when interspersed with heatwaves more accurately simulate the compounding stress these systems now face, with lasting effects on biomass recovery and soil microbial function (Hoover et al. 2014). In freshwater systems, press nutrient enrichment combined with heat stress can lead to persistent hypoxia and loss of biodiversity (Moss et al. 2011; Paerl and Paul 2012). Similarly, in marine environments, whereas pulse disturbances such as storm‐driven salinity changes can cause immediate physiological stress to intertidal organisms, press stressors like sustained warming or repeated microbial disruption can erode resilience over time, as presented here and within other marine systems (such as in corals, which bleach under recurrent thermal stress) (Hughes et al. 2017; Ziegler et al. 2019). These examples illustrate that climate change rarely delivers singular shocks, but rather, creates stress regimes that are both prolonged and punctuated by extreme events. Consequently, experimental approaches that layer pulse events onto press disturbances are increasingly necessary to understand—and accurately predict—the responses of organisms and ecosystems under future climate scenarios.

### 4.2 Effect of Microbial Disruption on Recovery of Host Function

Disruption to different components of the microbiota affected the host in different ways, an effect which was also induced by water of different salinities, a disruption which marine and estuarine organisms naturally experience (although typically not as severe as during this extreme event). Within the field, the observed changes in host physiology and reproduction occurred after the microbiota changed in response to our microbial disruption treatments, which is important because it may allow disentangling microbially mediated responses from just an experimental artefact, as any direct chemical effects of the antimicrobials used on the hosts or osmotic effects would likely be immediate (Davis et al. 2022; 2011). Furthermore, this temporal lag is consistent with natural systems where microbial communities often buffer short‐term stress but may collapse under chronic pressure. Press disruption with iodine inhibited the recovery of reproductive output for the entire experimental period (120 days). As in the pulse treatments, the main bacterial taxa that were reduced by the press treatment belonged to the class *Cyanophyceae*, but unlike the pulse treatments, the abundance of this taxa did not recover over the entire experimental period, which was correlated with no recovery of host reproductive output. These findings parallel field observations in which sustained disturbances, such as recurring heatwaves or extended freshwater exposure in estuaries, can erode microbial community resilience and lead to long‐term declines in host fitness (Shade et al. 2012; Ziegler et al. 2017). Framing microbial shifts within the pulse–press disturbance paradigm thus provides a powerful lens for understanding how escalating climate extremes may undermine organismal and ecosystem health through altered host–microbiome dynamics.

Disruption with antibiotics and iodine caused significant differences in the community structure of the microbiota when compared with controls. These differences were characterised by significant decreases in ASVs belonging to the class *Cyanophyceae*. The most abundant of these ASVs (*Pleurocaspa* sp) ranged from 3%–5% of the total community abundance, consistent with previous observations (McGrath et al. 2024). Cyanobacteria like *Pleurocaspa* sp., are suggested to be ubiquitous in the ocean and have been implicated in a number of symbioses with eukaryotic hosts (Schvarcz et al. 2022; Taylor 1973; Webster and Blackall 2009). As symbionts, Cyanobacteria have been reported to provide hosts with a number of key nutrients, including carbon (Foster et al. 2022), nitrogen (Fiore et al. 2010) and sulphur (Jensen et al. 2017). The provision of nutrients is key to host persistence, especially in nutrient‐limited areas such as the intertidal, grassland and aquatic environments (Adak et al. 2016; Bracken and Nielsen 2004; Elser et al. 2007; Talbot et al. 2018). Thus, disruption to the host's supply of nutrients by the loss of important microbial taxa potentially leads to lower reproductive output, as has been shown in plant systems (Y. Li et al. 2017; McGinley and Charnov 1988). Disrupting the microbiota of hosts with antibiotics has complex effects (Dittami et al. 2021; McGrath et al. 2024), making the determination of whether the observed effect on the host is through the direct effect of the treatment or microbially mediated challenging (Dittami et al. 2021; Marzinelli et al. 2024). The work presented here adds additional evidence to suggest the latter for *Hormosira*—that is, that recovery following environmental stress was a microbially mediated effect.

### 4.3 Short‐Term Performance Versus Long‐Term Fitness of Holobionts

Following the extreme rainfall event in March 2022, the photosynthetic efficiency of algae showed no changes through time and was similar to values reported for ‘healthy’ individuals of this species elsewhere (McGrath et al. 2024, 2025). Such physiological, or performance measures, which typically represent a small snapshot of an organism's life history, may not accurately represent long‐term performance of the host or its offspring, that is, fitness (Sebens et al. 2018). This appears to be the case here, given that reproductive output of individuals in the field changed in response to rainfall, but photosynthetic efficiency did not. It is therefore important to include measures with closer links to organisms' fitness, such as reproduction, to understand holobiont responses to, and recovery from, environmental stress. This is particularly important to understand for habitat‐forming holobionts, which form the biogenic structure of the ecosystems, as interactions within the holobiont can cascade throughout the entire ecosystem (Laforest‐Lapointe et al. 2017). Such understanding can also help identify mechanisms and determine the effect of specific microbial taxa on holobiont fitness, which may be important for developing novel tools to enhance resilience of habitat‐forming hosts to stress, for example, via microbiota engineering (Peixoto et al. 2021; Silverstein et al. 2023). Although our temporal patterns suggest that recovery following environmental stress was a microbially mediated effect, the specific microbial functions and mechanisms underpinning this require further exploration. Importantly, however, our experimental work indicates that repeated (press) disturbances of host microbiota prevented recovery of host reproductive output, which, given the increasing frequency of stress, can erode resilience and exacerbate population declines.

## Author Contributions

**Alexander H. McGrath:** conceptualization (lead), data curation (lead), formal analysis (lead), funding acquisition (supporting), investigation (lead), methodology (lead), project administration (equal), validation (equal), visualization (equal), writing – original draft (lead), writing – review and editing (lead). **Peter D. Steinberg:** conceptualization (equal), formal analysis (supporting), funding acquisition (equal), investigation (equal), methodology (equal), project administration (equal), resources (equal), supervision (lead), visualization (equal), writing – original draft (equal), writing – review and editing (equal). **Staffan Kjelleberg:** conceptualization (supporting), data curation (supporting), formal analysis (supporting), funding acquisition (equal), investigation (supporting), methodology (equal), project administration (equal), writing – original draft (equal), writing – review and editing (equal). **Ezequiel M. Marzinelli:** conceptualization (equal), data curation (equal), formal analysis (equal), funding acquisition (equal), investigation (equal), methodology (equal), project administration (equal), supervision (lead), visualization (equal), writing – original draft (equal), writing – review and editing (equal).

## Funding

This work was supported by the University of Sydney, William George Murrell bequest. Ecological Society of Australia, Holsworth Wildlife Research Endowment. Australian Research Council, DP180104041.

## Ethics Statement

The authors have nothing to report.

## Conflicts of Interest

The authors declare no conflicts of interest.

## Supporting information


**Data S1:** ece373644‐sup‐0001‐DataS1.zip.
**Figure S1:** Female *Hormosira* spawning in response to lowered salinity.
**Figure S2:** The structure of the bacterial community associated with *Hormosira* following microbial disruption in the field. Ordination on the basis of a GLLVM model with each panel indicating a different sampling day throughout the experiment, with *n* = 5 independent replicates of each of the 6 host treatments: Antibiotics (Red), Iodine pulse (Orange), Iodine press (Yellow), Control (Light blue), Press procedural control (Blue), Pulse procedural control (Navy blue).
**Figure S3:** Comparison of regressions examining the effect of March as a potential outlier.
**Table S1:** Analysis of monthly rainfall among months (fixed, 12 levels: January—December) (alpha = 0.05). Post hoc contrasts were calculated using the emmeans R function and *p*‐values adjusted for multiple‐testing.
**Table S2:** Specific dates each month the algae were collected and the analysis of the number of eggs released by *Hormosira* individuals each month (12 months) (alpha = 0.05). Post hoc contrasts were calculated using the emmeans R function and *p*‐values adjusted for multiple‐testing.
**Table S3:** Analysis of the number of eggs released by *Hormosira* individuals exposed to different salinities (fixed, 5 levels: 0, 5, 15, 25, and 35 ppt) (alpha = 0.05). Post hoc contrasts were calculated using the emmeans R function and *p*‐values adjusted for multiple‐testing.
**Table S4:** PERMANOVA on the basis of Bray–Curtis similarity measures for qPCR‐normalised, square‐root transformed bacterial ASV abundances on *Hormosira banksii* among different salinity treatments (fixed, 5 levels: 0, 5, 15, 25 and 35 ppt; *n* = 5) (alpha = 0.05). Post hoc contrasts were calculated using the pairwise. adonis R function and *p*‐values adjusted for multiple‐testing.
**Table S5:** PERMANOVA on the basis of Bray–Curtis similarity measures for qPCR‐normalised, square‐root transformed ASV abundances on *Hormosira banksii* among different microbial treatments (fixed, 6 levels: AB2, I, IP, C, PC, PPC) and sampling times (fixed, crossed, 7 levels over 120 days) (alpha = 0.05). Post hoc contrasts were calculated using the pairwise. Adonis R function and *p*‐values adjusted for multiple‐testing.
**Table S6:** Analysis of bacterial community alpha diversity measures: number of species and Simpson index in *Hormosira banksii* subject to different microbial treatments (fixed, 6 levels: AB2, I, IP, C, PC, PPC) and sampling times (fixed, crossed, 7 levels over 120 days) (alpha = 0.05). Post hoc pairwise comparisons calculated using emmeans.
**Table S7:** Analysis of host reproductive output among different microbial treatments (fixed, 6 levels: AB2, I, IP, C, PC, PPC) and sampling times (fixed, crossed, 7 levels over 120 days) (alpha = 0.05). Post hoc contrasts were calculated using the emmeans R function and *p*‐values adjusted for multiple‐testing.
**Table S8:** Analysis of numbers of developing eggs among different microbial treatments (fixed, 6 levels: AB2, I, IP, C, PC, PPC) and sampling times (fixed, crossed, 7 levels over 120 days) (alpha = 0.05). Post hoc contrasts were calculated using the emmeans R function and *p*‐values adjusted for multiple‐testing.
**Table S9:** Analysis of host photosynthetic yield among different microbial treatments (fixed, 6 levels: AB2, I, IP, C, PC, PPC) and sampling times (fixed, crossed, 7 levels over 120 days) (alpha = 0.05). Post hoc contrasts were calculated using the emmeans R function and *p*‐values adjusted for multiple‐testing.

## Data Availability

Data and standard code are provided as via the following link https://doi.org/10.5281/zenodo.17020491. Raw sequences are available on NCBI under accession number PRJNA1444916.
