# Supplementary material for: Effects of Extreme Rainfall on a Dominant Seaweed Are Mitigated by Its Microbiota
Source: Ecol Evol. 2026 May 14;16(5):e73644. doi: 10.1002/ece3.73644 (PMC13174576; doi:10.1002/ece3.73644)
Supplement: Supplementary file 1 — Data S1: ece373644‐sup‐0001‐DataS1.zip. Figure S1: Female Hormosira spawning in response to lowered salinity. Figure S2: The structure of the bacterial community associated with Hormosira following microbial disruption in the field. Ordination on the basis of a GLLVM model with each panel indicating a different sampling day throughout the experiment, with n = 5 independent replicates of each of the 6 host treatments: Antibiotics (Red), Iodine pulse (Orange), Iodine press (Yellow), Control (Light blue), Press procedural control (Blue), Pulse procedural control (Navy blue). Figure S3: Comparison of regressions examining the effect of March as a potential outlier. Table S1: Analysis of monthly rainfall among months (fixed, 12 levels: January—December) (alpha = 0.05). Post hoc contrasts were calculated using the emmeans R function and p‐values adjusted for multiple‐testing. Table S2: Specific dates each month the algae were collected and the analysis of the number of eggs released by Hormosira individuals each month (12 months) (alpha = 0.05). Post hoc contrasts were calculated using the emmeans R function and p‐values adjusted for multiple‐testing. Table S3: Analysis of the number of eggs released by Hormosira individuals exposed to different salinities (fixed, 5 levels: 0, 5, 15, 25, and 35 ppt) (alpha = 0.05). Post hoc contrasts were calculated using the emmeans R function and p‐values adjusted for multiple‐testing. Table S4: PERMANOVA on the basis of Bray–Curtis similarity measures for qPCR‐normalised, square‐root transformed bacterial ASV abundances on Hormosira banksii among different salinity treatments (fixed, 5 levels: 0, 5, 15, 25 and 35 ppt; n = 5) (alpha = 0.05). Post hoc contrasts were calculated using the pairwise. adonis R function and p‐values adjusted for multiple‐testing. Table S5: PERMANOVA on the basis of Bray–Curtis similarity measures for qPCR‐normalised, square‐root transformed ASV abundances on Hormosira banksii among different microbial treatmen [file ECE3-16-e73644-s001.zip › ece373644-sup-0001-Supinfo.docx]

Supplementary information - Effects of extreme rainfall on a dominant seaweed are mitigated by its microbiota

Alexander H. McGrath^1,2,*^, Peter D. Steinberg^2,3^, Staffan Kjelleberg^4^, Ezequiel M. Marzinelli^1,2,4^

^1^The University of Sydney, School of Life and Environmental Sciences, Sydney, New South Wales, Australia

^2^ Sydney Institute of Marine Science, Mosman, New South Wales, Australia

^3^ Centre for Marine Science and Innovation, School of Biological, Earth, and Environmental Science, University of New South Wales, Sydney, New South Wales, Australia

^4^ Singapore Centre for Environmental Life Sciences Engineering, Nanyang Technological University, 60 Nanyang Drive, SBS-01N-27, Singapore 637551, Republic of Singapore

*Corresponding author. Current address: Via Derna 1, Pisa 56126, Italy Email: [alexander.mcgrath@biologia.unipi.it](mailto:alexander.mcgrath@biologia.unipi.it)

Short title: Effect of extreme rainfall on a coastal holobiont

Keywords: climate change, holobiont, extreme weather, microbiome, reproduction, antimicrobial treatments, macroalga

### DNA Extraction and sequencing

Microbial DNA was extracted from each swab sample in a randomised order to avoid introducing any bias due to order and time of processing, using a Powersoil DNA Isolation kit (Qiagen) following the manufacturers protocol. DNA extracts were quantified using spectrophotometry (Nanodrop 1000) and stored at -20oC until sequencing.

The extracted DNA samples were amplified with Polymerase Chain Reaction (PCR) using the 16S primers 341 (F) (5’- CCTACGGGNGGCWGCAG-3’) and 805(R) – (5’-GACTACHVGGGTATCTAATCC-‘3), containing the V3-V4 regions of the bacterial and archaeal 16S rRNA gene (Klindworth et al., 2013). The PCR conditions involved a pre-heating step to 95 °C for 3 min followed by 35 cycles of 95 °C for 15 s, 55 °C for 1 min and 73 °C for 30 s. Both positive (with known DNA sequence) and negative controls (nuclease-free water, control swabs) were used. The negative controls did not amplify DNA, suggesting no contamination on swabs or during extraction and amplification. Agarose gel electrophoresis and Nanodrop 1000 were used to ensure the quantity and quality of the amplicons before they were sent for sequencing via the Illumina MiSeq 2000 platform at the Ramaciotti Centre for Genomics (UNSW, Sydney).

### Bioinformatics

UNOISE was then used to remove chimeras and produce amplicon sequence variants (ASVs), i.e., amplicon sequence variants at a unique sequence level (0% distance) (Edgar, 2016). DADA2 was used to map the original reads back to ASVs, generating a table of 17,387 ASVs. ASV sequences were searched with BlastN against the SILVA SSU Ref NR99 database for taxonomic classification to classify and remove chloroplasts; Global Taxonomy Database (GTDB) was then used for taxonomic assignment. Singletons and low abundance taxa (<0.01% of reads) were removed from the dataset for statistical analyses, resulting in 13,210 microbial taxa.

### Estimation of absolute bacterial abundance

Total abundance of the 16S rRNA gene was quantified for each sample by qPCR using the primers 341F/805R developed by (Thijs et al., 2017). Gene amplification and analysis were performed using the QuantStudio 3 thermocycler (Thermo Fisher with PrimeTime® Gene Expression Master Mix, Integrated DNA Technologies) and associated software. The reaction conditions for amplification of DNA were 50^o^C for 2 min, 95 ^o^C for 10 min and 40 cycles of 95 ^o^C for 15s and 60 ^o^C for 1 min. The final gene copy number per sample was corrected for the total extraction volume, the surface area and the dilution factor and DNA yield per sample (see Nappi et al 2021 for further details) and were used to estimate absolute abundances of ASVs and inoculants.

**Statistical analyses**

***Rainfall***

To test for extreme events, defined as rare events (>95^th^ probability percentile) for the area and time of year (IPCC 2023), each month in 2022 was ranked against the previous 50 years of rainfall data, on a month by month basis. To determine which months (within 2022) had the greatest amount of daily rainfall, we used a one factor ANOVA with the fixed factor of month (12 levels). Furthermore, to determine how salinity changed during extreme rainfall events, salinity was taken from the Bio Oracle V3 database and ranked against previous years (Assis et al. 2024). Rainfall in Sydney during 2022 overall was 209% greater than the average of all years since data has been collected (164 years). In March, rainfall was significantly higher than all other months in 2022, with the highest daily rainfall on the 3^rd^ of March (153mm; Figure 1a). We characterised the rainfall in March 2022 as an extreme event as the rainfall was greater than the 99^th^ percentile of monthly rainfall on record (Malan et al. 2024) with June and October both being present within the 90th percentile.

**Field Experiment**

To test for effects of microbial disruption on host performance and reproduction in the field experiment (PAM, number of developing eggs in *Hormosira* fronds, number of eggs released), we used a two-factor ANOVA with the orthogonal factors treatment (fixed, 6 levels) and time (fixed, 7 levels) using the R GAD package. To meet the model’s assumption of homogeneity of variance, the response variables a) number of developing eggs and b) eggs released were square-root transformed. Post-hoc contrasts were run on significant interaction terms, or significant main effects (when no interactions were present) with more than two levels, using emmeans in R (Lenth et al. 2019).

**Bacterial analyses**

For bacterial data, to account for uneven sequencing depth among samples, data were normalised using total 16S rRNA reads per sample of the v3-v4 region which was calculated using qPCR (Nappi et al. 2022). Alpha diversity measures of richness (i.e. number of unique sequences) and Simpson’s diversity index were calculated using the ‘vegan’ R package (Oksanen et al. 2013). and differences between treatments (fixed, 6 levels), time (fixed, crossed, 7 levels) and their interaction were examined using an ANOVA in the R GAD package (Sandrini-Neto et al. 2010) as described above. For the salinity lab experiment, bacterial data were processed and analysed in the same way but with the single factor salinity (fixed, 5 levels).

To determine differences in the structure of the host-associated bacterial assemblages, the normalised ASV data were analysed using permutational multivariate analysis of variance (Anderson and Walsh 2013) in the R vegan package (Oksanen et al. 2013). with the fixed factors treatment, time and their interaction (for the field experiment), or with the fixed factor salinity (for the lab experiment), as above. These multivariate analyses were based on Bray-Curtis dissimilarities between sample pairs calculated on square-root transformed absolute (qPCR normalised) abundances of ASVs. For significant main effects or significant interactions, pairwise comparisons were performed using the pairwise.adonis.2 function in the pairwise.adonis package (Martinez Arbizu 2020).

To determine which bacterial taxa differed the most among treatments and times and their interaction (for the field experiment), or among salinity levels (for the lab experiment), we used multivariate generalised linear models (GLMs) using the R package ‘mvabund’(Wang et al. 2012) assuming a negative binomial distribution to account for over-dispersion of sequence counts. Generalised linear latent variable models (GLLVMs) were used to visualise the bacterial community structure(Niku et al. 2019).


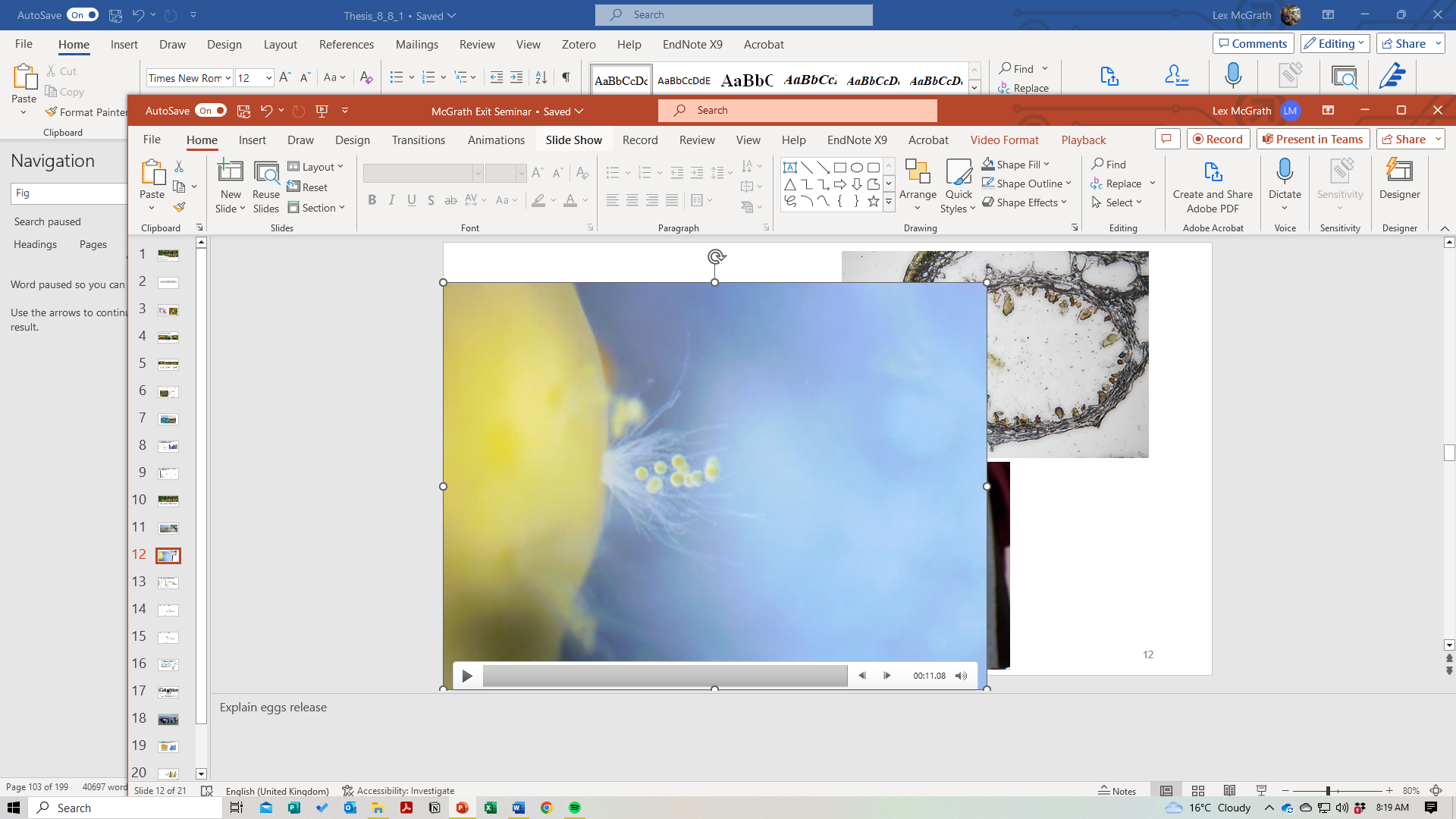


Supplementary Figure 1. Female *Hormosira* spawning in response to lowered salinity.


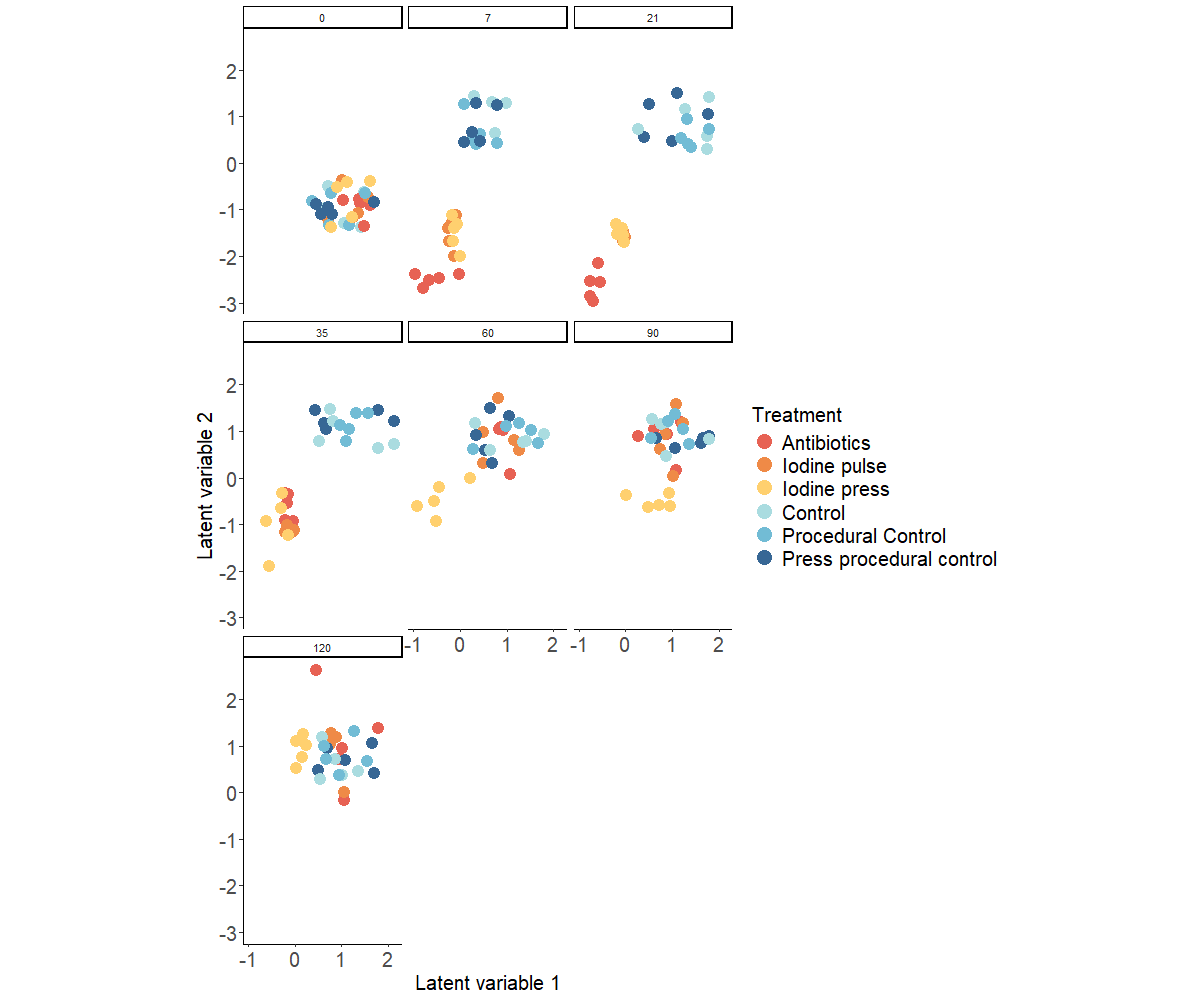
Supplementary figure 2 **The structure of the bacterial community associated with *Hormosira* following microbial disruption in the field**. Ordination based on a GLLVM model with each panel indicating a different sampling day throughout the experiment, with n=5 independent replicates of each of the 6 host treatments: Antibiotics (Red), Iodine pulse (Orange), Iodine press (Yellow), Control (Light blue), Press procedural control (Blue), Pulse procedural control (Navy blue).


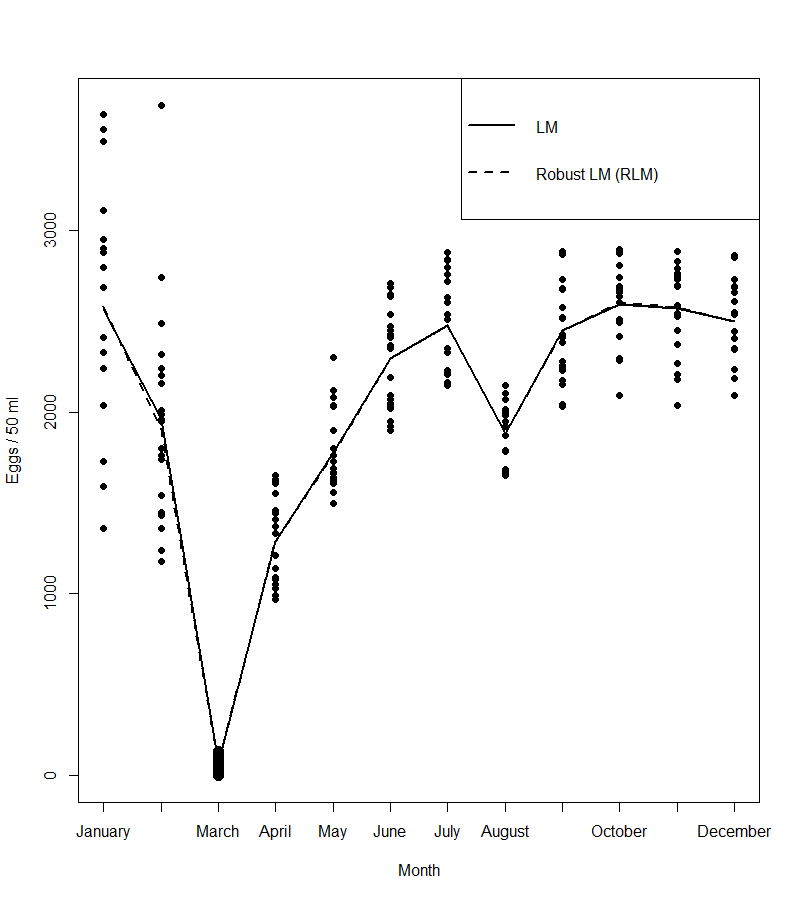


Supplementary figure 3 Comparison of regressions examining the effect of March as a potential outlier.

| **Supplementary Table 1**. Analysis of monthly rainfall among months (fixed, 12 levels: January – December)(alpha=0.05). Post-hoc contrasts were calculated using the emmeans R function and p-values adjusted for multiple-testing. | | | | | | |
| --- | --- | --- | --- | --- | --- | --- |
| Source |  | df | Sum of Sq | Mean Sq | F | p |
| Month |  | 11 | 6332 | 575.6 | 431.8 | <0.001 |
| Residual |  | 551 | 384 | 1.3 |  |  |
|  |  |  |  |  |  |  |
| Pairwise contrasts |  |  |  |  |  |  |
| Month | March > February > July =October >January = April = June = August = September = November = December | | | | | |

| **Supplementary Table 2**. Specific dates each month the algae was collected and the Analysis of number of eggs released by *Hormosira* individuals each month (12 months) (alpha=0.05). Post-hoc contrasts were calculated using the emmeans R function and p-values adjusted for multiple-testing. | | | | | | | | | | | | |
| --- | --- | --- | --- | --- | --- | --- | --- | --- | --- | --- | --- | --- |
| Specific dates | |  | |  | |  | |  | |  | |  |
| Month | | Date | | Month | | Date | | Month | | Date | |  |
| October | | 19/10/21 | | April | | 14/4/22 | | October | | 13/10/22 | |  |
| November | | 11/11/21 | | May | | 19/5/22 | | November | | 14/11/22 | |  |
| December | | 14/12/21 | | June | | 9/6/22 | | December | | 9/12/22 | |  |
| January | | 11/1/22 | | July | | 12/7/22 | | January | | 16/1/23 | |  |
| February | | 16/2/22 | | August | | 23/8/22 | | February | | 14/2/23 | |  |
| March | | 24/3/22 | | September | | 6/9/22 | | March | | 10/3/23 | |  |
|  | |  | |  | |  | |  | |  | |  |
|  | |  | |  | |  | |  | |  | |  |
| Source | |  | | Df | | Sum of Sq | | Mean Sq | | F | | p |
| Month | |  | | 11 | | 144565 | | 36141 | | 374.8 | | <0.001 |
| Residual | |  | | 228 | | 43163 | | 9592 | |  | |  |
|  | |  | |  | |  | |  | |  | |  |
| Pairwise comparison | |  | |  | |  | |  | |  | |  |
| Month | | January>February>March<April<May>June=July>August<September=October=November=December | | | | | | | | | | |
| contrast | | estimate | | SE | | df | | t.ratio | | p.value | |  |
| April - August | | -595.30 | | 103.887 | | 228 | | -5.73027 | | 0.00000 | |  |
| April - December | | -1,214.70 | | 103.887 | | 228 | | -11.69252 | | 0.00000 | |  |
| April - Febuary | | -676.00 | | 103.887 | | 228 | | -6.50707 | | 0.00000 | |  |
| April - January | | -1,282.00 | | 103.887 | | 228 | | -12.34034 | | 0.00000 | |  |
| April - July | | -1,192.95 | | 103.887 | | 228 | | -11.48315 | | 0.00000 | |  |
| April - June | | -1,010.00 | | 103.887 | | 228 | | -9.72211 | | 0.00000 | |  |
| April - March | | 1,224.00 | | 103.887 | | 228 | | 11.78204 | | 0.00000 | |  |
| April - May | | -490.00 | | 103.887 | | 228 | | -4.71667 | | 0.00026 | |  |
| April - November | | -1,283.95 | | 103.887 | | 228 | | -12.35911 | | 0.00000 | |  |
| April - October | | -1,308.85 | | 103.887 | | 228 | | -12.59879 | | 0.00000 | |  |
| April - September | | -1,161.45 | | 103.887 | | 228 | | -11.17994 | | 0.00000 | |  |
| August - December | | -619.40 | | 103.887 | | 228 | | -5.96225 | | 0.00000 | |  |
| August - Febuary | | -80.70 | | 103.887 | | 228 | | -0.77681 | | 0.99977 | |  |
| August - January | | -686.70 | | 103.887 | | 228 | | -6.61007 | | 0.00000 | |  |
| August - July | | -597.65 | | 103.887 | | 228 | | -5.75289 | | 0.00000 | |  |
| August - June | | -414.70 | | 103.887 | | 228 | | -3.99184 | | 0.00493 | |  |
| August - March | | 1,819.30 | | 103.887 | | 228 | | 17.51230 | | 0.00000 | |  |
| August - May | | 105.30 | | 103.887 | | 228 | | 1.01360 | | 0.99728 | |  |
| August - November | | -688.65 | | 103.887 | | 228 | | -6.62884 | | 0.00000 | |  |
| August - October | | -713.55 | | 103.887 | | 228 | | -6.86852 | | 0.00000 | |  |
| August - September | | -566.15 | | 103.887 | | 228 | | -5.44967 | | 0.00001 | |  |
| December - Febuary | | 538.70 | | 103.887 | | 228 | | 5.18544 | | 0.00003 | |  |
| December - January | | -67.30 | | 103.887 | | 228 | | -0.64782 | | 0.99996 | |  |
| December - July | | 21.75 | | 103.887 | | 228 | | 0.20936 | | 1.00000 | |  |
| December - June | | 204.70 | | 103.887 | | 228 | | 1.97041 | | 0.71287 | |  |
| December - March | | 2,438.70 | | 103.887 | | 228 | | 23.47455 | | 0.00000 | |  |
| December - May | | 724.70 | | 103.887 | | 228 | | 6.97585 | | 0.00000 | |  |
| December - November | | -69.25 | | 103.887 | | 228 | | -0.66659 | | 0.99995 | |  |
| December - October | | -94.15 | | 103.887 | | 228 | | -0.90627 | | 0.99901 | |  |
| December - September | | 53.25 | | 103.887 | | 228 | | 0.51258 | | 1.00000 | |  |
| Febuary - January | | -606.00 | | 103.887 | | 228 | | -5.83326 | | 0.00000 | |  |
| Febuary - July | | -516.95 | | 103.887 | | 228 | | -4.97608 | | 0.00008 | |  |
| Febuary - June | | -334.00 | | 103.887 | | 228 | | -3.21503 | | 0.06445 | |  |
| Febuary - March | | 1,900.00 | | 103.887 | | 228 | | 18.28911 | | 0.00000 | |  |
| Febuary - May | | 186.00 | | 103.887 | | 228 | | 1.79041 | | 0.82169 | |  |
| Febuary - November | | -607.95 | | 103.887 | | 228 | | -5.85203 | | 0.00000 | |  |
| Febuary - October | | -632.85 | | 103.887 | | 228 | | -6.09172 | | 0.00000 | |  |
| Febuary - September | | -485.45 | | 103.887 | | 228 | | -4.67287 | | 0.00031 | |  |
| January - July | | 89.05 | | 103.887 | | 228 | | 0.85718 | | 0.99941 | |  |
| January - June | | 272.00 | | 103.887 | | 228 | | 2.61823 | | 0.27584 | |  |
| January - March | | 2,506.00 | | 103.887 | | 228 | | 24.12237 | | 0.00000 | |  |
| January - May | | 792.00 | | 103.887 | | 228 | | 7.62367 | | 0.00000 | |  |
| January - November | | -1.95 | | 103.887 | | 228 | | -0.01877 | | 1.00000 | |  |
| January - October | | -26.85 | | 103.887 | | 228 | | -0.25845 | | 1.00000 | |  |
| January - September | | 120.55 | | 103.887 | | 228 | | 1.16040 | | 0.99130 | |  |
| July - June | | 182.95 | | 103.887 | | 228 | | 1.76105 | | 0.83707 | |  |
| July - March | | 2,416.95 | | 103.887 | | 228 | | 23.26519 | | 0.00000 | |  |
| July - May | | 702.95 | | 103.887 | | 228 | | 6.76649 | | 0.00000 | |  |
| July - November | | -91.00 | | 103.887 | | 228 | | -0.87595 | | 0.99928 | |  |
| July - October | | -115.90 | | 103.887 | | 228 | | -1.11564 | | 0.99374 | |  |
| July - September | | 31.50 | | 103.887 | | 228 | | 0.30321 | | 1.00000 | |  |
| June - March | | 2,234.00 | | 103.887 | | 228 | | 21.50414 | | 0.00000 | |  |
| June - May | | 520.00 | | 103.887 | | 228 | | 5.00544 | | 0.00007 | |  |
| June - November | | -273.95 | | 103.887 | | 228 | | -2.63700 | | 0.26563 | |  |
| June - October | | -298.85 | | 103.887 | | 228 | | -2.87668 | | 0.15650 | |  |
| June - September | | -151.45 | | 103.887 | | 228 | | -1.45783 | | 0.95012 | |  |
| March - May | | -1,714.00 | | 103.887 | | 228 | | -16.49870 | | 0.00000 | |  |
| March - November | | -2,507.95 | | 103.887 | | 228 | | -24.14114 | | 0.00000 | |  |
| March - October | | -2,532.85 | | 103.887 | | 228 | | -24.38083 | | 0.00000 | |  |
| March - September | | -2,385.45 | | 103.887 | | 228 | | -22.96198 | | 0.00000 | |  |
| May - November | | -793.95 | | 103.887 | | 228 | | -7.64244 | | 0.00000 | |  |
| May - October | | -818.85 | | 103.887 | | 228 | | -7.88212 | | 0.00000 | |  |
| May - September | | -671.45 | | 103.887 | | 228 | | -6.46327 | | 0.00000 | |  |
| November - October | | -24.90 | | 103.887 | | 228 | | -0.23968 | | 1.00000 | |  |
| November - September | | 122.50 | | 103.887 | | 228 | | 1.17917 | | 0.99007 | |  |
| October - September | | 147.40 | | 103.887 | | 228 | | 1.41885 | | 0.95879 | |  |

| **Supplementary Table 3**. Analysis of number of eggs released by *Hormosira* individuals exposed to different salinities (fixed, 5 levels: 0, 5, 15, 25, 35 ppt) (alpha=0.05). Post-hoc contrasts were calculated using the emmeans R function and p-values adjusted for multiple-testing. | | | | | | |
| --- | --- | --- | --- | --- | --- | --- |
| Source |  | Df | Sum of Sq | Mean Sq | F | p |
| Salinity |  | 4 | 144565 | 36141 | 374.8 | <0.001 |
| Residual |  | 45 | 43163 | 9592 |  |  |
|  |  |  |  |  |  |  |
| Pairwise comparison |  |  |  |  |  |  |
| Salinity | 0<5<15<25=35 |  |  |  |  |  |
| contrast | estimate | SE | df | t.ratio | p.value |  |
| Salinity0 - Salinity5 | 450.2 | 38.06435 | 45 | 11.82734 | 0.00000 |  |
| Salinity0 - Salinity15 | 1,303.1 | 38.06435 | 45 | 34.23413 | 0.00000 |  |
| Salinity0 - Salinity25 | 1,359.7 | 38.06435 | 45 | 35.72108 | 0.00000 |  |
| Salinity0 - Salinity35 | 1,372.2 | 38.06435 | 45 | 36.04947 | 0.00000 |  |
| Salinity5 - Salinity15 | 852.9 | 38.06435 | 45 | 22.40679 | 0.00000 |  |
| Salinity5 - Salinity25 | 909.5 | 38.06435 | 45 | 23.89375 | 0.00000 |  |
| Salinity5 - Salinity35 | 922.0 | 38.06435 | 45 | 24.22214 | 0.00000 |  |
| Salinity15 - Salinity25 | 56.6 | 38.06435 | 45 | 1.48696 | 0.00057 |  |
| Salinity15 - Salinity35 | 69.1 | 38.06435 | 45 | 1.81535 | 0.00037 |  |
| Salinity25 - Salinity35 | 12.5 | 38.06435 | 45 | 0.32839 | 0.99739 |  |

| **Supplementary** **Table 4**. PERMANOVA based on Bray–Curtis similarity measures for qPCR-normalised, square-root transformed bacterial ASV abundances on *Hormosira banksii* among different salinity treatments (fixed, 5 levels: 0, 5, 15, 25 and 35ppt; n=5) (alpha = 0.05). Post-hoc contrasts were calculated using the pairwise.adonis R function and p-values adjusted for multiple-testing. | | | | | | |
| --- | --- | --- | --- | --- | --- | --- |
| Source | df | SS | MS | pseudo-*F* | *p*(perm) | Number of permutations |
| Salinity | 4 | 514 | 152 | 3.87 | <0.001 | 9946 |
| Residual | 45 | 641 | 284 |  |  |  |
| Total | 49 | 1352 |  |  |  |  |
| Pairwise comparisons | 0 different to 5 different to 15 different to 25=35 |  |  |  |  |  |

| **Supplementary Table 5**. PERMANOVA based on Bray–Curtis similarity measures for qPCR-normalised, square-root transformed ASV abundances on *Hormosira banksii* among different microbial treatments (fixed, 6 levels: AB2, I, IP, C, PC, PPC) and sampling times (fixed, crossed, 7 levels over 120 days) (alpha = 0.05). Post-hoc contrasts were calculated using the pairwise.adonis R function and p-values adjusted for multiple-testing. | | | | | | |
| --- | --- | --- | --- | --- | --- | --- |
| Source | df | SS | MS | pseudo-*F* | *p*(perm) | perms |
| Treatment | 5 | 4499.3 | 1795.8 | 1.988 | <0.001 | 9698 |
| time | 6 | 94683 | 6487.7 | 4.2726 | <0.001 | 9891 |
| Treatment x time | 30 | 145271 | 26814.9 | 6.903 | <0.002 | 9787 |
| Residual | 168 | 60587 | 682.19 |  |  |  |
| Total | 209 | 305040.3 |  |  |  |  |
| Pairwise comparisons |  |  |  |  |  |  |
|  | Day | Treatment |  |  |  |  |
|  | 0 | AB2=I=IP=C=PC=PPC |  |  |  |  |
|  | 7 | AB2 different from I=IP different from C=PC=PPC |  |  |  |  |
|  | 21 | AB2 Different from I=IP different from C=PC=PPC |  |  |  |  |
|  | 35 | AB2=I=IP different from C=PC=PPC |  |  |  |  |
|  | 60 | IP different from AB2=I=C=PC=PPC |  |  |  |  |
|  | 90 | IP different from AB2=I=C=PC=PPC |  |  |  |  |
|  | 120 | IP different from AB2=I=C=PC=PPC |  |  |  |  |
|  | 7=21 different from 35 different from 0=60=90=120 | AB2 |  |  |  |  |
|  | 7 different from 21=35<0=60=90=120 | I |  |  |  |  |
|  | 90 different from 7=60=120 different from 21=35 different from 0 | IP |  |  |  |  |
|  | 0 different from 7 different from 21=35=60=90=120 | C |  |  |  |  |
|  | 0 different from 7 different from 21=35=60=90=120 | PC |  |  |  |  |
|  | 0 different from 7 different from 21=35=60=90=120 | PPC |  |  |  |  |

| **Supplementary Table 6** Analysis of bacterial community alpha diversity measures: number of species and Simpson index in *Hormosira banksii* subject to different microbial treatments (fixed, 6 levels: AB2, I, IP, C, PC, PPC) and sampling times (fixed, crossed, 7 levels over 120 days) (alpha = 0.05). Post-hoc pairwise comparisons calculated using emmeans. | | | | | | |
| --- | --- | --- | --- | --- | --- | --- |
| *Number of Species* |  |  |  |  |  |  |
| Source |  | Df | Sum of Sq | Mean Sq | F | P-value |
| Treatment |  | 5 | 101780 | 20356 | 4.9231 | <0.0001 |
| Time |  | 6 | 143095 | 35774 | 8.6519 | <0.0001 |
| Treatment x Time |  | 30 | 69236 | 3462 | 0.8372 | 0.6632 |
| Residual |  | 168 | 372129 | 4135 |  |  |
|  |  |  |  |  |  |  |
| Pairwise comparisons |  |  |  |  |  |  |
| Day | 0=7<21<35=60=90=120 |  |  |  |  |  |
| Treatment | AB2=IP<I<C=PC=PPC |  |  |  |  |  |
|  |  |  |  |  |  |  |
| *Simpsons Index* |  |  |  |  |  |  |
| Source |  | Df | Sum of Sq | Mean Sq | F | P-value |
| Treatment |  | 5 | 92483 | 21463 | 5.213 | <0.0001 |
| Time |  | 6 | 159346 | 32856 | 9.213 | <0.0001 |
| Treatment x Time |  | 30 | 72564 | 4286 | 0.6136 | 0.6632 |
| Residual |  | 168 | 385641 | 5143 |  |  |
|  |  |  |  |  |  |  |
| Pairwise comparisons |  |  |  |  |  |  |
| Day | 0=7<21<35=60=90=120 |  |  |  |  |  |
| Treatment | AB2=IP<I<C=PC=PPC |  |  |  |  |  |
|  |  |  |  |  |  |  |
